# Evaluation of population-based screening programs on colorectal cancer screening uptake and predictors in Atlantic Canada: insights from a repeated cross-sectional study

**DOI:** 10.1186/s44263-024-00061-6

**Published:** 2024-05-06

**Authors:** Kazeem Adefemi, John C. Knight, Yun Zhu, Peizhong Peter Wang

**Affiliations:** 1 Division of Population Health and Applied Health Sciences, Faculty of Medicine, Memorial University of Newfoundland, St. John’s, NL A1B 3V6 Canada; 2 The Beatrice Hunter Cancer Research Institute, Dalhousie University, Halifax, NS Canada; 3 Data and Information Services, NL Health Services, St. John’s, NL Canada; 4 Centre de Recherche du CHUM, Montreal, QC Canada; 5 Dalla Lana School of Public Health, University of Toronto, Toronto, ON Canada

**Keywords:** Colorectal cancer, Cancer screening, Healthcare disparities, Health determinants, Cancer prevention, Atlantic Canada

## Abstract

**Background:**

Colorectal cancer (CRC) poses a significant public health challenge in Canada, with the Atlantic provinces bearing a particularly high burden. The implementation of population-based colon screening programs is aimed to address this concern. However, limited research exists on the effect of these programs especially in Canada. This study aimed to examine the impact of the first few years of the CRC screening programs in the Atlantic provinces of Canada by assessing changes in screening uptake, barriers, and predictors of screening among eligible populations.

**Methods:**

Employing a repeated cross-sectional design, this study analyzed data from a representative sample of 7614 respondents in 2010 and 6850 in 2017 from the Atlantic provinces aged 50–74 years, extracted from the Canadian Community Health Survey (CCHS). The outcomes measured were CRC screening rates, changes in predictors of screening uptake, and barriers to participation. Potential predictors examined included age, sex, income, education, smoking, and health status.

**Results:**

The proportion of adults aged 50–74 years who meet CRC screening requirements increased from 42% in 2010 to 54% in 2017 yet below the national target of 60%. New Brunswick reported the most significant increase in screening prevalence (18%, *p* < 0.05). Participation in fecal tests increased from 19.6 to 32.4%. Despite these improvements, disparities in screening participation remained, with lower uptake observed among individuals with lower income and education levels. Age (> 60 years, *OR* = 2.09, *p* < 0.01), the presence of multiple chronic health conditions (*OR* = 2.11, *p* < 0. 01), being female (*OR* = 1.21, *p* < 0.01), married status (*OR* = 1.21, *p* < 0.05), access to regular healthcare (*OR* = 1.91, *p* < 0.01), and nonsmoking status (*OR* = 2.55, *p* < 0.01) were identified as significant predictors of CRC screening uptake.

**Conclusions:**

This study shows that while CRC screening uptake increased across the Atlantic provinces between 2010 and 2017, barriers to and disparities in screening participation persist. This highlights the need for targeted interventions to improve awareness, access, and screening uptake, particularly among disadvantaged groups, to promote equitable healthcare outcomes. Continued efforts should focus on reducing barriers to screening and leveraging available evidence to inform interventions aimed at mitigating the CRC burden in the region.

## Background

Despite being one of the few cancers that can be effectively prevented, the health, financial, and health resources burden of colorectal cancer (CRC) in Canada remains a growing and significant public health concern [1]. CRC ranks as the third most diagnosed cancer and the second leading cause of cancer-related mortality, with 24,300 new cases and 9400 associated mortalities in 2022 alone [2, 3]. The burden of CRC is particularly concerning in the Atlantic provinces of Canada [3]. With age-standardized incidence and mortality rates ranging from 102.9 to 42.4 in Newfoundland and Labrador to 62.1 and 26.7 in New Brunswick respectively, CRC incidence and mortality are highest among all age groups in the Atlantic provinces compared to the rest of Canada [4, 5].

Screening plays a crucial role in reducing the incidence and mortality of CRC, and numerous studies have demonstrated the importance and effectiveness of various screening modalities in this regard [6, 7]. Although the Atlantic provinces of Canada established population-based CRC screening programs between 2009 and 2014 [2] (see Table 1), the region continues to experience higher rates of CRC incidence and mortality compared with other parts of Canada [8].

**Table 1:** Details of provincial CRC screening program in Atlantic Canada [2]

Province	Program name	Program start date; status	Recruitment/invitation method	Screening test details	Normal result communication & recall
New Brunswick (NB)	NB Colon Cancer Screening Program	2014; full program, province wide	Mailed invitation letter	FIT^a^ every 2 years^b^, from 50–74 years	Result mailed to participant, re-invitation and eligibility questionnaire sent after 2 years
Nova Scotia (NS)	Colon Cancer Prevention Program	2009; full program, province wide	Mailed invitation letter and FIT kit sent after 2 weeks	FIT every 2 years, from 50–74 years	Result mailed to participant, recall reminder and kit mailed every 2 years
Prince Edward Island (PEI)	Colorectal Cancer Screening Program	2011; full program, province wide	Mailed invitation letter, physician referral, self-referral by phone, email, online, or in person	FIT every 2 years, from 50–75 years	Result mailed to participant recall reminder and kit mailed every 2 years. In-person *kit* pick-up available
Newfoundland and Labrador (NL)	NL Colon Cancer Screening Program	2012; full program, province wide	Physician referral; self-referral by phone, email, or in person; referral through other screening program; website	FIT every 2 years, from 50–74 years	Result mailed to participant, recall reminder mailed every 2 years

^a^*FIT* fecal immunochemical test for “asymptomatic, average risk” individuals

^b^All provincial colon cancer screening programs are designed in line with guidelines from the *Canadian Task Force on Preventive Health Care*, that is, CRC screening for individuals at average risk between the ages of 50 and 74, with a fecal test every 2 years or flexible sigmoidoscopy/colonoscopy test every 10 years. There are two recommended fecal tests — guaiac fecal occult blood test (gFOBT) and fecal immunochemical test (FIT). CRC screening programs in the Atlantic provinces have used FIT since inception

Organized, public health screening programs offer distinct advantages over opportunistic screening, as they improve awareness about the importance of screening for cancer prevention and address barriers and inequities in screening access [6]. Evaluating the impact of these screening programs is essential for assessing changes in CRC screening behaviors and uptake and identifying areas that might require further intervention to improve screening participation [4]. Moreover, given the higher burden of CRC in the Atlantic provinces, optimizing the outcomes of CRC screening programs is particularly crucial [9]. Unfortunately, research evaluating the impact of these programs has been limited. This study, using data from 2010 and 2017 due to the availability of comparable, comprehensive CRC screening data for all Atlantic provinces, provides an assessment of CRC screening uptake after the implementation of screening programs in the region. This study seeks to inform policymakers, healthcare providers, and public health practitioners about the effectiveness of current screening strategies and the need for interventions to address unique challenges faced by the Atlantic provinces. The goal is to contribute actionable insights that can lead to improved screening uptake, reduced CRC incidence, and ultimately, enhanced health outcomes for populations in these regions.

## Methods

### Study design

In this study, we employed a repeated cross-sectional design, which involved secondary analysis of cross-sectional data from the 2010 and 2017 cycles of the CCHS [10, 11]. Unlike longitudinal studies that follow the same individuals over time, repeated cross-sectional studies analyze data from different samples at multiple time points. This approach allows for the examination of trends and changes in population-level outcomes [12], such as CRC screening uptake.

### Data source

The data for this study was obtained from the master files of 2010 and 2017 CCHS, a national cross-sectional survey conducted by Statistics Canada [10, 11], Canada’s national statistical agency (Ottawa, Canada). The survey collects detailed information on health status, healthcare utilization, sociodemographic details, and health determinants. The CCHS interviews about 65,000 people aged 12 years and above, annually, from all health regions of Canada, excluding full-time members of the armed forces, and individuals living in reserves and some remote communities (less than 3% of the population) [11]. The CCHS uses a multistage, cluster sampling technique to ensure the representativeness of the sample and collected data.

For this study, the data of respondents aged 50–74 years in the Atlantic provinces (New Brunswick, Newfoundland & Labrador, Nova Scotia, and Prince Edward Island) were analyzed. Total analytic sample from the Atlantic provinces was 7614 (weighted *n* = 1,449,028) and 6850 (weighted *n* = 1,472,700) respectively for 2010 and 2017. Although the CCHS underwent a major redesign in 2013, and Statistics Canada advises against merging pre- and post-2015 files for analyses [11], comparing estimates from 2010 and 2017, analyzed separately, provides valuable insight into changes in utilization of crucial health services such as cancer screening.

### Outcome and predictor variables

The study focused on two main outcome variables: CRC screening history (referred to as “ever-screen”) and being up-to-date with CRC screening (referred to as “screen up-to-date”). Ever-screen was defined as a history of exposure to any CRC screening tests, while screen up-to-date was defined as participation in a fecal test within the 2 years or an endoscopy test within the 10 years before the survey. In the questionnaire, the 2017 CCHS distinguished between sigmoidoscopy and colonoscopy, but this distinction was absent in 2010. To ensure consistency, responses from both years were aggregated under the broader term “endoscopy test.”

The study evaluated various sociodemographic and health behavior factors associated with CRC and/or CRC screening uptake in previous studies. The sociodemographic variables assessed included age (categorized into 5-year age groups), sex, marital status, education, and total household income. Additionally, self-reported health status (five categories in the CCHS aggregated into poor, good, and great), access to a regular healthcare provider, obesity (using body mass index (BMI), international standard), number of comorbidities, smoking status, and physical activity level were assessed as potential predictors. The 2017 CCHS collected data on barriers to screening among respondents who reported no history of CRC screening, providing some qualitative insights into factors influencing screening behaviors.

### Statistical analyses

All analyses were weighted and bootstrapped using survey weights and 500 replicate bootstrap sampling weights provided by Statistics Canada. This weighting and bootstrapping ensured that the estimates were representative of the general population and accounted for the complex survey sampling design. Only weighted proportions are reported to comply with Statistics Canada’s confidentiality and data protection requirements.

Descriptive analyses were conducted using survey procedures in SAS 9.4 (SAS Institute Inc. 2013) to evaluate the distribution of sociodemographic and health behavior characteristics. Proportions of respondents with a history of CRC screening and those up-to-date with CRC screening were estimated for the years 2010 and 2017, by screening modality (fecal or endoscopy), and by province. Bivariate analyses were performed to determine differences in screening prevalence across different sociodemographic groups.

Logistic regression analyses were conducted to assess the association between the predictor variables and CRC screening uptake. Initially, logistic analyses were performed with each potential predictor variable, adjusting for age and sex a priori. Subsequently, a fully saturated multivariate logistic regression model was developed, including all covariates except education level due to its strong correlation with income. Stratified analyses by sex were also conducted to assess whether the predictors of screening uptake significantly varied between males and females. The results are reported as weighted proportions (%) and odds ratios (OR) with associated 95% confidence intervals (CI).

## Results

### Demographic characteristics

This study analyzed data from a sample of 7614 respondents in 2010 (weighted *n* = 1,449,028) and 6850 respondents in 2017 (weighted *n* = 1,472,700). Table 2 presents a demographic breakdown of the sample. In both 2010 and 2017, there were slightly more females (51%) than males on average. An overall aging trend was observed in the four provinces, with a decrease in the proportion of people in their 50 s (from 48 to 41%) and an increase in the proportion of people in their 60 s (39 to 44%) and 70 s (13 to 15%). The prevalence of obesity and multimorbidity increased, while the income gap widened over the study period. The proportion of people with a household income of at least US $80,000 almost doubled from 21 to 41%, while those with a household income of US $40,000 or less decreased from 32 to 26%.

**Table 2:** Demographic characteristics of respondents in 2010 and 2017

Characteristics	Atlantic provinces, % of population									
	New Brunswick (NB)		Newfound & Labrador (NL)		Nova Scotia (NS)		Prince Edward Island (PEI)		Atlantic average	
	2010 (%) Weighted *n* = 237,836	2017 (%) Weighted *n* = 260,620	2010 (%) Weighted *n* = 167,397	2017 (%) Weighted *n* = 194,962	2010 (%) Weighted *n* = 291,758	2017 (%) Weighted *n* = 332,344	2010 (%) Weighted *n* = 42,726	2017 (%) Weighted *n* = 51,684	2010 (%) Weighted *n* = 739,718	2017 (%) Weighted *n* = 839,611
**Sex**										
Female	51.0	51.2	52.1	50.3	53.1	52.1	50.1	50.2	51.6	51.0
Male	49.0	48.8	47.9	49.7	46.9	47.9	49.9	49.8	48.4	49.0
**Age**										
50**–**54	24.8	17.5	23.1	21.0	25.0	18.4	22.0	21.1	23.7	19.5
55**–**59	26.4	23.1	23.6	19.6	23.9	23.0	22.9	21.6	24.2	21.8
60**–**64	21.0	26.6	23.9	23.7	21.2	24.0	24.4	22.0	22.6	24.1
65**–**69	16.0	18.2	17.4	23.0	17.7	18.5	16.1	18.8	16.8	19.6
70**–**74	11.9	14.5	12.0	12.8	12.2	16.2	14.6	16.5	12.7	15.0
**Marital Status**										
Married, common law	76.3	74.2	80.2	73.7	76.1	73.5	79.2	74.9	77.9	74.1
Single, never married	7.3	7.6	5.2	7.2	5.5	9.4	5.8	9.2	6.0	8.4
Widow, separated, divorced	16.4	18.1	14.7	19.0	18.3	17.1	14.8	15.8	16.0	17.5
Missing	0.0	0.0	0.0	0.1	0.1	0.0	0.2	0.1	0.1	0.0
**Education**										
Post Sec. Sch	54.0	50.5	51.4	53.0	62.8	59.0	60.8	54.7	57.2	54.3
Sec. Sch	19.7	28.7	14.2	21.6	11.8	22.0	10.7	19.5	14.1	22.9
Less Sec. Sch	21.3	19.0	32.7	24.5	23.7	15.8	28.3	23.7	26.5	20.8
Missing	5.0	1.7	1.7	0.9	1.7	3.2	0.3	2.2	2.2	2.0
**Household Income**										
US $100 k +	14.5	29.7	12.8	30.8	17.4	33.7	14.8	28.1	14.9	30.6
US $80,000–US $99,999	5.8	12.8	6.0	7.6	5.9	11.6	7.0	11.4	6.2	10.9
US $60,000–$79,999	14.5	13.9	10.1	14.4	13.5	14.6	14.5	15.0	13.1	14.5
US $40,000**–**US $59,999	17.8	17.2	19.9	18.5	17.3	16.6	19.4	20.2	18.6	18.1
*Less* US $39,999	33.3	26.4	37.1	28.6	30.4	23.5	28.8	25.3	32.4	25.9
Missing	14.2		14.1		15.5		15.5		14.8	
**Self-reported health status**										
Excellent	45.4	47.1	55.1	55.9	48.4	51.9	63.1	56.7	53.0	52.9
Good	47.8	47.8	39.2	39.6	44.8	41.9	29.5	37.3	40.3	41.6
Poor	6.8	4.8	5.4	4.5	6.9	5.7	7.3	5.9	6.6	5.2
**Weight**^**a**^										
Normal	28.3	21.1	26.9	14.7	31.1	21.5	33.4	25.5	29.9	20.7
Obese	30.6	41.9	30.3	39.4	26.6	36.6	26.1	32.4	28.4	37.6
Overweight	34.4	31.8	37.9	38.6	37.7	36.5	36.6	36.5	36.7	35.9
Underweight	1.0	1.0	0.3	1.4	1.0	0.7	1.1	0.2	0.8	0.8
Missing	5.7	4.2	4.8	5.9	3.7	4.7	2.8	5.4	4.2	5.0
**Number of comorbidities**										
0	23.2	16.4	19.8	14.7	21.2	17.8	27.3	25.2	22.9	18.5
1**–**2	44.7	45.6	45.8	48.3	46.0	46.2	44.8	40.5	45.3	45.1
3**–**5	25.1	31.1	29.6	31.6	28.0	30.7	23.5	30.2	26.6	30.9
6 +	7.0	7.0	4.8	5.3	4.8	5.3	4.4	4.1	5.2	5.4
**Has regular care provider**	93.8	96.2	93.8	90.5	95.7	91.5	92.3	87.1	93.9	91.3
**Physical activity level**										
Active	20.3	32.9	16.5	36.4	24.2	33.7	18.1	32.9	19.8	34.0
Moderate	24.2	31.9	23.6	32.5	23.0	36.4	25.0	37.0	24.0	34.4
Inactive	52.3	30.0	56.7	27.0	51.0	26.1	55.0	26.5	53.7	27.4
**Smoking**										
Daily	17.3	9.7	18.1	21.1	18.9	15.4	14.9	16.9	17.3	15.8
Occasional	2.3	2.7	2.2	2.9	2.6	3.6	6.5	3.2	3.4	3.1
Nonsmoker	80.1	87.6	79.2	75.9	78.2	80.9	78.7	79.9	79.1	81.1

^a^Weight categories based on BMI international standards. *Sec. Sch* Secondary school

### Screening participation

Between 2010 and 2017, the Atlantic provinces recorded a notable increase in the prevalence of screening history for colorectal cancer, with rates rising from 53 to 67%, representing a 14% increase in the proportion of people who have been exposed to any CRC screening test (Table 3). Among the four provinces, New Brunswick (NB) saw the biggest change in proportion of people with CRC screening history, from 50 to 70%. Newfoundland and Labrador (NL), which initially had one of the highest screening rates in 2010, reported the lowest change from 54.5 to 62.8%. Similar proportions were observed in individuals with up-to-date CRC screening, increasing from 42 to 54.5%, on average. However, this fell short of the national target of 60% screening participation rate. NB recorded the biggest change in up-to-date CRC screening, from 39 to 57%, second only to Nova Scotia (NS) with a screening rate of 59.6%. These two provinces saw statistically significant change in the proportion of people up-to-date with CRC screening. In contrast, NL reported a 5% change in screening rates. The examination of screening test preferences shows a nuanced shift in line with the emphasis on fecal test as the primary screening test promoted through CRC screening programs. While uptake of endoscopy tests declined slightly, this was accompanied by a significant increase in fecal tests participation from an average of 19.6% in 2010 to 32.4% in 2017. This shift varied by province, however, with the highest change of 21 to 44% in NS and the lowest of 18 to 23% in NL.

**Table 3:** CRC screening participation by province in 2010 and 2017

Screening tests	Atlantic provinces; % of population									
	NB		NL		NS		PEI		Atlantic average	
	2010	2017	2010	2017	2010	2017	2010	2017	2010	2017
Any exposure to CRC screening test — “**ever-screen**”	**50.4**	**70.3**	**54.5**	**62.8**	**53.8**	**69.7**	**54.8**	**66.6**	**53.4**	**67.3**
Fecal test < 2 years	13.8	28.6	18.9	23.0	21.3	44.3	24.5	33.7	19.6	32.4
Endoscopy test < 10 years	25.2	28.3	25.0	25.8	21.2	15.3	17.8	18.8	22.3	22.1
Either/both — **Screen_up-to-date**	**39.0**	**56.9**	**43.9**	**48.8**	**42.5**	**59.6**	**42.3**	**52.5**	**41.9**	**54.5**
Change, 2010 to 2017 (%)	17.9*		**4.9**		17.1*		**10.2**		**12.6**	

*NB* New Brunswick, *NL* Newfoundland & Labrador, *NS* Nova Scotia, *PEI* Prince Edward Island

^*^Significant at *p* > *0.05*

Table 4 illustrates the demographic distribution of respondents up-to-date with CRC screening. In 2010, 53% of women were up-to-date with CRC screening, compared to 47% of men; by 2017, women’s participation slightly decreased to 52%, while men’s participation increased to 48%. This change shows a modest convergence in screening rates between the sexes over the study period, except for PEI, where men reported a higher screening participation rate of 54% in 2017. Further, on average, while screening participation increased among people in their 60 s and 70 s by 3% and 2%, respectively, from 2010 to 2017, it decreased by 5% among people in their 50s.

**Table 4:** Distribution of (up to date) CRC screening participation by demographic characteristics

Demographic characteristics	Atlantic provinces; % of population									
	NB		NL		NS		PEI		Atlantic average	
	2010	**2017**	2010	**2017**	2010	**2017**	2010	**2017**	2010	**2017**
**Sex**										
Female	51.7	**51.0**	53.3	**57.0**	56.0	**54.9**	51.3	**46.0**	53.1	**52.2**
Male	48.3	**49.0**	46.7	**43.0**	44.0	**45.1**	48.7	**54.0**	46.9	**47.8**
**Age**										
50–54	17.3	**14.4**	23.5	**18.8**	15.9	**13.7**	15.1	**14.1**	18.0	**15.3**
55–59	24.7	**19.5**	20.6	**16.3**	25.9	**22.5**	18.4	**22.0**	22.4	**20.1**
60–64	23.4	**30.7**	25.0	**26.9**	23.6	**26.4**	30.8	**25.2**	25.7	**27.3**
65–69	20.5	**18.6**	17.6	**24.0**	20.3	**18.8**	17.7	**20.3**	19.0	**20.4**
70–74	14.1	**16.8**	13.3	**14.0**	14.3	**18.6**	18.1	**18.5**	14.9	**17.0**
**Marital status**										
Married, common law	79.4	**75.3**	79.2	**72.2**	79.5	**76.1**	83.7	**79.3**	80.4	**75.7**
Single, never married	4.9	**5.7**	4.6	**7.5**	2.8	**7.9**	2.7	**6.8**	3.7	**7.0**
Widow, separated, divorced	15.8	**19.1**	16.2	**20.3**	17.6	**16.0**	13.6	**13.9**	15.8	**17.3**
**Education**										
Post sec. sch	58.3	**50.1**	58.6	**57.0**	64.4	**61.9**	67.2	**57.7**	62.1	**56.7**
Sec. sch	14.5	**28.1**	15.8	**20.7**	11.4	**20.1**	6.7	**21.8**	12.1	**22.7**
Less sec. sch	23.9	**20.2**	24.5	**21.3**	22.7	**15.0**	25.4	**19.8**	24.1	**19.1**
Missing data	3.2	**1.6**	1.1	**1.0**	1.5	**3.0**	0.7	**0.8**	1.6	**1.6**
**Household income**										
US $80 k +	34.7	**41.1**	32.1	**40.6**	38.8	**46.4**	39.6	**41.3**	36.3	**42.4**
US $40 k–US $80 k	30.4	**30.8**	32	**30.4**	32.3	**32.4**	33.9	**37.9**	32.1	**32.9**
< US $40 k	34.9	**28.1**	35.9	**29.1**	28.9	**21.2**	26.6	**20.7**	31.6	**24.8**
**Number of comorbidities**										
0	15.3	**11.3**	14.0	**6.1**	15.4	**13.4**	26.6	**22.2**	17.8	**13.3**
1–2	45.0	**52.6**	44.8	**52.1**	41.8	**46.0**	43.1	**47.8**	43.7	**49.6**
3–5	30.0	**27.8**	36.9	**35.3**	36.2	**34.9**	25.5	**26.9**	32.1	**31.3**
6 +	9.8	**8.3**	4.3	**6.5**	6.6	**5.7**	4.8	**3.0**	6.4	**5.9**

In 2010, screening participation was highest among married individuals, people with income above US $80,000, and those with postsecondary school education. While this pattern remains consistent in 2017, there was decline in screening rates among married individuals (80 to 76%) and people with postsecondary education (62 to 57%) but increase screening among people who earn US $80,000 + (36 to 42%). Between 2010 and 2017, CRC screening participation almost doubled among people who report a high school education (12 to 23%).

### Reasons for and barriers to CRC screening

Table 5 presents the self-reported barriers to participating in CRC screening. Among individuals with no screening history, approximately 41% and 50% did not participate because they deemed the fecal and endoscopy tests, respectively, to be unnecessary. Additionally, 24% and 37% did not participate because their healthcare provider considered the fecal and endoscopy tests to be unnecessary.

**Table 5:** Self-reported barriers to CRC screening — 2017

**Variable**	**% of respondents who had no fecal test in preceding 2 years**				
	**NB**	**NL**	**NS**	**PEI**	**Atlantic average**
Did not know about the test	5.0	2.3	0.6	0.6	2.1
Doctor says test not necessary	29.9	33.3	13.6	18.8	23.9
Fear/discomfort	1.1	0.4	4.6	0.3	1.6
Had endoscopy test instead	10.9	11.6	14.1	14.6	12.8
Lack of time	1.9	0.6	9.3	7.3	4.8
No access to test	1.4	1.5	0.5	0.2	0.9
No doctor	0.3	0.1	2.2	0.3	0.7
Other	11.3	4.9	17.8	15.4	12.3
Did not think test is necessary	38.1	45.4	37.2	42.4	40.8
**Variable**	**% of respondents who had no endoscopy test in preceding 10 years**				
Did not know about the test	0.4	0.0	0.3	1.1	0.5
Doctor says test not necessary	43.1	37.0	33.2	35.5	37.2
Fear/discomfort	1.7	1.3	1.9	0.0	1.3
Had *fecal* test	1.3	1.0	4.2	2.6	2.3
Lack of time	0.3	0.4	2.1	0.1	0.7
No access to test	0.4	1.1	0.2	1.4	0.8
No doctor	0.3	0.1	1.5	0.4	0.6
Other	9.5	3.8	7.3	4.7	6.3
Did not think test is necessary	43.0	55.2	49.2	54.2	50.4

### Predictors of screening

Building on the demographic distribution of CRC screening participation outlined in the preceding section, our multivariate logistic regression analysis adjusted for potential covariates for 2010 and 2017 revealed consistent predictors of screening participation across both years (Table 6). For instance, those in their 60 s (*AOR* 1.95 95% *CI* 1.39–2.73, 2010, and 2.09 95% *CI* 1.49–2.94, 2017) or 70 s (*AOR* 2.20 95% *CI* 1.51–3.20, 2010, and 1.96 95% *CI* 1.32–2.92, 2017), married (1.51 95% *CI* 1.04–2.18, 2010, and 1.21 95% *CI* 0.82–1.77, 2017), having multiple chronic health conditions (2.69 95% *CI* 1.91–3.78, 2010, and 2.11 95% *CI* 1.50–2.96, 2017), and having a regular healthcare provider (2.27 95% *CI* 1.32–3.89, 2010, and 1.91 95% *CI* 1.30–2.80, 2017) were consistently associated with increased screening likelihood. Conversely, daily smokers, people who are single, obese individuals, and those reporting excellent health (0.83 95% *CI* 0.50–1.37, 2010, and 0.99 95% *CI* 0.57–1.73, 2017) had decreased screening odds. In 2010, low income, especially household income below US $40,000, was linked to lower screening odds, but this was no longer significant in 2017. In 2017, being male (0.79 95% *CI* 0.64–0.99) and residing in Newfoundland and Labrador were associated with decreased screening odds. When stratified by sex, the predictors of screening uptake were similar but slightly stronger among men.

**Table 6:** Predictors of up-to-date CRC screening in Atlantic provinces in 2010 and 2017, stratified by sex

Variable	2010			2017		
	Adjusted OR (95% CI)			Adjusted OR (95% CI)		
	Overall	Men	Women	Overall	Men	Women
**Age**						
50–54	1.0 (ref)**	1.0 (ref)	1.0 (ref)**	1.0 (ref)**	1.0 (ref)**	1.0 (ref)
55–59	1.46 (1.04–2.07)	1.25 (0.72–2.18)	1.68 (1.09–2.59)	1.29 (0.92–1.82)	1.40 (0.79–2.48)	1.21 (0.76–1.95)
60–64	1.95 (1.39–2.73)	1.56 (0.95–2.57)	2.47 (1.59–3.82)	2.09 (1.49–2.94)	3.31 (1.88–5.83)	1.50 (0.90–2.49)
65–69	1.93 (1.36–2.73)	1.46 (0.86–2.50)	2.56 (1.65–3.99)	1.56 (1.09–2.23)	1.83 (1.04–3.21)	1.37 (0.82–2.29)
70–74	2.20 (1.51–3.20)	2.21 (1.23–3.95)	2.26 (1.37–3.72)	1.96 (1.32–2.92)	1.88 (0.97–3.63)	2.23 (1.30–3.83)
**Sex**						
F	1.0 (ref)			1.0 (ref)**		
M	0.97 (0.76–1.22)			0.79 (0.64–0.99)		
**Comorbidity**						
0	1.0 (ref)**	1.0 (ref)**	1.0 (ref)**	1.0 (ref)**	1.0 (ref)**	1.0 (ref)**
1–2	1.50 (1.13–1.99)	1.64 (1.06–2.56)	1.49 (1.01–2.26)	2.30 (1.69–3.12)	1.99 (1.31–3.03)	2.56 (1.62–4.05)
3–5	2.69 (1.91–3.78)	2.39 (1.31–4.37)	3.25 (2.03–5.21)	2.11 (1.50–2.96)	1.94 (1.21–3.11)	2.30 (1.35–3.92)
6 +	3.38 (1.91–5.98)	1.60 (0.48–5.30)	5.27 (2.46–11.27)	2.81 (1.62–4.87)	4.50 (1.94–10.43)	2.00 (1.01–4.48)
**Province**						
NFLD	1.0 (ref)	1.0 (ref)	1.0 (ref)	1.0 (ref)**	1.0 (ref)**	1.0 (ref)
NB	0.83 (0.65–1.06)	0.93 (0.60–1.45)	0.76 (0.54–1.07)	1.36 (1.01–1.83)	1.47 (0.96–2.24)	1.15 (0.76–1.74)
NS	0.90 (0.70–1.16)	0.80 (0.53–1.22)	0.96 (0.67–1.39)	1.54 (1.21–1.96)	1.61 (1.07–2.42)	1.43 (1.00–2.07)
PEI	0.87 (0.58–1.32)	0.86 (0.41–1.82)	0.92 (0.57–1.49)	1.52 (1.09–2.14)	2.4 (1.4–4.12)	1.05 (0.63–1.71)
**Marital status**						
Single, never married	1.0 (ref)**	1.0 (ref)**	1.0 (ref)	1.0 (ref)**	1.0 (ref)	1.0 (ref)
Married, common law	1.51 (1.04–2.18)	2.49 (1.35–4.62)	0.97 (0.57–1.66)	1.21 (0.82–1.77)	1.27 (0.77–2.11)	1.24 (0.66–2.33)
Widow, separated, divorced	1.40 (0.94–2.11)	2.04 (1.09–3.84)	1.03 (0.56–1.88)	1.15 (0.77–1.72)	1.21 (0.70–2.11)	1.16 (0.63–2.14)
**Household income**						
< US $39,999	1.0 (ref)*	1.0 (ref)	1.0 (ref)	1.0 (ref)	1.0 (ref)	1.0 (ref)
US $40,000–59,999	1.09 (0.74–1.58)	0.76 (0.46–1.30)	1.378 (0.90–2.12)	0.99 (0.73–1.35)	0.73 (0.44–1.19)	1.19 (0.75–1.88)
US $60,000–79,999	1.40 (0.99–1.98)	1.10 (0.63–1.92)	1.83 (1.12–2.99)	1.00 (0.717–1.39)	0.99 (0.58–1.68)	0.93 (0.57–1.51)
US $80,000–99,999	0.98 (0.61–1.59)	0.78 (0.40–1.52)	1.21 (0.56–2.62)	0.79 (0.54–1.173)	0.7 (0.37–1.32)	0.73 (0.43–1.23)
US $100 k and over	1.42 (0.93–2.16)	0.99 (0.53–1.85)	1.89 (1.04–3.43)	1.14 (0.87–1.59)	1.01 (0.59–1.71)	1.18 (0.75–1.84)
**Perceived health status**						
Poor	1.0 (ref)**	1.0 (ref)**	1.0 (ref)	1.0 (ref)	1.0 (ref)	1.0 (ref)
Good	0.88 (0.56–1.40)	0.66 (0.29–1.48)	1.30 (0.70–2.40)	1.11 (0.66–1.86)	1.02 (0.48–2.19)	1.36 (0.65–2.85)
Excellent	0.83 (0.50–1.37)	0.54 (0.23–1.29)	1.36 (0.72–2.60)	0.99 (0.57–1.73)	0.72 (0.33–1.59)	1.36 (0.57–3.2)
**Weight#**						
Obese	1.0 (ref)**	1.0 (ref)**	1.0 (ref)	1.0 (ref)**	1.0 (ref)**	1.0 (ref)
Overweight	1.43 (1.11–1.86)	1.81 (1.22–2.69)	1.19 (0.82–1.72)	1.10 (0.86–1.42)	1.25 (0.85–1.82)	0.97 (0.65–1.45)
Normal	1.27 (0.92–1.70)	1.19 (0.73–1.91)	1.38 (0.90–2.11)	1.07 (0.79–1.44)	1.13 (0.70–1.83)	1.05 (0.69–1.59)
Underweight	1.07 (0.44–2.57)	1.53 (0.18–13.03)	0.85 (0.29–2.53)	0.97 (0.17–5.68)	2.81 (0.03–27.48)	0.45 (0.03–6.20)
**Regular healthcare provider**						
No	1.0 (ref)**	1.0 (ref)**	1.0 (ref)	1.0 (ref)**	1.0 (ref)**	1.0 (ref)
Yes	2.27 (1.32–3.89)	3.04 (1.50–6.16)	1.61 (0.70–3.71)	1.91 (1.30–2.80)	2.33 (1.39–3.90)	1.58 (0.82–3.03)
**Physical activity level**						
Inactive	1.0 (ref)**	1.0 (ref)**	1.0 (ref)**	1.0 (ref)	1.0 (ref)	1.0 (ref)*
Moderate activity	1.25 (0.96–1.64)	1.516 (1.00–2.30)	1.05 (0.73–1.52)	1.09 (0.82–1.45)	1.14 (0.73–1.76)	1.18 (0.78–1.79)
Rigorous activity	1.18 (0.86–1.58)	1.29 (0.84–1.98)	1.11 (0.73–1.67)	1.20 (0.90–1.59)	1.05 (0.69–1.60)	1.60 (1.10–2.35)
**Smoking status**						
Daily	1.0 (ref)**	1.0 (ref)**	1.0 (ref)	1.0 (ref)**	1.0 (ref)**	1.0 (ref)**
Occasional	1.68 (0.85–3.34)	2.01 (0.67–5.99)	1.41 (0.52–3.85)	1.23 (0.64–2.37)	0.75 (0.25–2.28)	1.76 (0.68–4.54)
Never smoked	1.68 (1.25–2.25)	1.64 (1.04–2.57)	1.60 (1.09–2.36)	2.55 (1.95–3.33)	2.62 (1.71–3.99)	2.58 (1.75–3.82)
**Fruits & vegetable consumption**						
< 5 serve daily	1.0 (ref)	1.0 (ref)	1.0 (ref)	1.0 (ref)**	1.0 (ref)	1.0 (ref)
5–10 serve daily	0.99 (0.79–1.26)	0.99 (0.65–1.52)	1.02 (0.74–1.41)	0.97 (0.73–1.29)	1.01 (0.64–1.60)	1.01 (0.70–1.45)
10 + serve daily	3.01 (1.14–7.94)	3.10 (0.31–30.86)	3.06 (1.19–7.89)	5.19 (1.79–15.06)	0.74 (0.02–25.89)	13.05 (0.04–99.99)

**p > .001; *p > .05. **#**Weight categories based on BMI international standards

## Discussion

Our study assessed colorectal cancer (CRC) screening uptake in the Atlantic provinces of Canada — NB, NL, NS, and PEI — comparing before and after the implementation of organized provincial CRC screening programs in line with national guidelines [13]. Our findings indicate that while screening participation increased post-implementation of these programs, the magnitude and nature of this change varied across provinces. Persistent disparities in CRC screening participation, particularly among certain demographic groups, were evident.

The data indicates differing change in CRC screening uptake across the Atlantic provinces between 2010 and 2017. NS and NB approached the national CRC screening target of 60%, while NL and PEI showed more modest gains within the same period. This variability observed among provinces underscores the multifaceted determinants of health service utilization. For instance, the duration of the CRC screening programs did not seem to determine the magnitude of change in screening uptake observed. NB with a relatively recent screening program reported a more pronounced change in screening uptake than NL and PEI. Instead, aspects such as promotional and recruitment strategies, coupled with broader socio-economic factors, could hold more influence [9, 14]. Such findings warrant a deeper examination of the specific strategies employed by each province, allowing for a cross-provincial learning where effective strategies could be shared and adapted.

Nonetheless, the magnitude of change in screening uptake is comparable to the effect of organized screening programs reported in other jurisdictions, such as in Ontario, Canada [15], the UK [16], France [17], and Spain [18, 19]. Despite the improvements in screening uptake, our study found persistent inequalities in CRC screening participation across the Atlantic provinces, particularly related to age, income, education levels, and health status.

These disparities in CRC screening uptake, especially among younger adults (50 s) and those facing socio-economic disadvantages, are particularly concerning. The younger demographic stands to potentially benefit more from early cancer detection [20]. So, any decline in screening uptake among this group demands attention. Furthermore, the disparities related to socio-economic factors reflect the broader global health challenge of ensuring equitable access to health resources and the multifaceted factors that influence (preventive) health decisions and behaviors [9, 21]. Such disparities are not just numbers; they represent lives, many of which could be saved with early detection.

Predictors of CRC screening participation remained largely consistent across both 2010 and 2017 and aligned with evidence from similar studies [22–26]. Age, marital status, income, education levels, and health behaviors such as smoking are consistently associated with screening behaviors. Achieving equitable access and participation across socioeconomic groups is one of the main goals of population-wide screening programs [27]. However, the provincial CRC screening programs in Atlantic Canada have yet to fully achieve this objective. Targeted interventions addressing barriers specific to different age, sex, and socioeconomic groups are necessary to address these disparities and ensure higher CRC screening participation [28].

Qualitative data from the 2017 CCHS provided insights into people’s awareness, beliefs, and attitudes toward CRC screening in the Atlantic provinces. A notable segment of respondents deemed the CRC test unnecessary or reportedly felt discouraged by their healthcare providers. This is in line with previous research regarding awareness and attitudes toward CRC screening [29]. Given the higher prevalence of CRC risk factors, incidence, and mortality in the Atlantic provinces [1, 3, 8], addressing these (mis)perceptions and attitudes is crucial. Frameworks like health belief model offer structured strategies to address such challenges. By emphasizing the severity of CRC and the crucial role of early detection, public health initiatives could potentially alter these perceptions [30, 31].

In light of our findings of persistent disparities in CRC screening participation, it is imperative to address these inequalities with multicomponent interventions that have shown promise in various jurisdictions and should be applicable in the Atlantic provinces [9, 14, 27]. These strategies should not only cater to the diverse needs of different demographic groups but also aim to address challenges specific to age, sex, and socioeconomic status. Our study underscores the importance of continuous evaluation and adaptation of screening programs to meet the evolving health landscape of the Atlantic provinces.

### Limitations

The self-reported nature of the data collected for the CCHS surveys introduces the possibility of participants inaccurately recalling their CRC screening history. While Nova Scotia’s CRC screening program was established in 2009, 1 year before our study’s baseline of 2010, data on CRC screening for all provinces in the Atlantic region are only available for 2010 and 2017. Notably, the 2018 to 2022 CCHS did not collect CRC screening data from provinces in the region. Further, we are aware that implementation of the CRC screening program is unlikely to be the sole causal factor for changes observed in screening uptake, given the multitude of factors that affect screening behaviors. However, despite these limitations, we believe that using data from the 2010 and 2017 CCHS surveys allows for an initial assessment of the impact CRC screening programs on screening uptake and identification of predictors of screening. Another limitation is the insufficient data within the CCHS to exclude individuals that fall outside the “average risk” eligibility requirement for the screening programs. However, these individuals are estimated to constitute less than 3% of the general population. Overall, our study provides a necessary review of the impacts and limitations of the first few years of provincial CRC screening programs in Atlantic Canada.

## Conclusions

This study highlights the positive contribution of provincial CRC screening programs to participation rates across the Atlantic provinces, though with notable inter-provincial variations. Persistent disparities in screening participation exist within provinces, particularly affecting people in their 50 s and socio-economically disadvantaged groups. These findings emphasize the need for targeted interventions to promote equitable access, address misconceptions through community-based initiatives and tailored messaging, and facilitate cross-provincial collaboration for best practice sharing. Improving overall screening rates and achieving equity in access remain critical public health priorities for reducing the burden of CRC throughout the Atlantic provinces.

## Data Availability

No datasets were generated or analysed during the current study.

## Abbreviations

CRC: Colorectal cancer
CCHS: Canadian Community Health Survey
CTFPHC: Canadian Task Force on Preventive Health Care
FIT: Fecal immunochemical test
FOBT: Fecal occult blood test
NB: New Brunswick
NL: Newfoundland & Labrador
NS: Nova Scotia
PEI: Prince Edward Island
RDC: Research Data Centre
